# Assessment of Cholesterol, Glycemia Control and Short- and Long-Term Antihypertensive Effects of Smooth Hound Viscera Peptides in High-Salt and Fructose Diet-Fed Wistar Rats

**DOI:** 10.3390/md17040194

**Published:** 2019-03-27

**Authors:** Ola Abdelhedi, Hana Khemakhem, Rim Nasri, Mourad Jridi, Leticia Mora, Ikram Ben Amor, Kamel Jamoussi, Fidel Toldrá, Jalel Gargouri, Moncef Nasri

**Affiliations:** 1 Laboratory of Enzyme Engineering and Microbiology, National School of Engineering of Sfax (ENIS), University of Sfax, P.O. Box 1173, Sfax 3038, Tunisia; ryma47@hotmail.fr (R.N.); jridimourad@gmail.com (M.J.); mon_nasri@yahoo.fr (M.N.); 2 Laboratory of Biochemistry, CHU HediChaker, University of Sfax, Sfax 3000, Tunisia; hanakhemakhem@gmail.com (H.K.); jamoussikamel@yahoo.fr (K.J.); 3 Higher Institute of Biotechnology of Monastir, University of Monastir, Monastir 5000, Tunisia; 4 Higher Institute of Biotechnology of Beja, University of Jendouba, Beja 9000, Tunisia; 5 Instituto de Agroquímica y Tecnologíade Alimentos (CSIC), Avenue Agustín Escardino 7, Paterna, 46980 Valencia, Spain; lemoso@iata.csic.es (L.M.); ftoldra@iata.csic.es (F.T.); 6 Centre Régional de Transfusion Sanguine de Sfax, Route El-Ain Km 0.5, Sfax 3003, Tunisia; ikbeam@yahoo.fr (I.B.A.); jalelgargouri@yahoo.fr (J.G.)

**Keywords:** smooth hound peptide, hypertension, anticoagulant activity, lipase inhibitory activity, high salt and fructose diet

## Abstract

In this study, the antihypertensive activity of Purafect^®^-smooth hound viscera protein hydrolysate (VPH) and its peptide fraction with molecular weight (MW) below 1 kDa (VPH-I) was investigated. In addition, the lipase inhibitory activity, as well the anticoagulant potential, in vitro, were assessed. The antihypertensive effects of VPH and VPH-I were studied during 24 h (short-term effect) and 30 days (long-term effect) using high-salt (18% NaCl) and -fructose (10%) diet (HSFD)-induced hypertension. Data showed that, 4 h post-administration of VPH and VPH-I (200 mg/kg BW), the systolic blood pressure of rats was reduced by about 6 and 9 mmHg, respectively. These effects were similar to that obtained with Captopril (~9 mmHg at t = 4 h). On the other hand, exposing the rats to daily to HSFD, coupled to the administration of viscera peptides, was found to attenuate hypertension. In addition, the proteins’ treatments were able to correct lipid and glycemic disorders, by reducing the total cholesterol and triglyceride contents and resorting to the plasma glucose level, compared to the HSFD group. Overall, the present findings demonstrated the preventive effect of VPH-peptides from hypertension complications, as a result of their biological properties.

## 1. Introduction

Fructose consumption has been dramatically increased during the recent decades, mainly because of the increase production of fructose-containing products [1]. Nevertheless, it was established that a high-fructose diet is associated with numerous health issues, like obesity [2], type II-diabetes [3], insulin resistance [4], renal failure [5], and hypertension. Indeed, Klein and Kiat [6] have shown that high-fructose diet up-regulates sodium (Na) and chloride (Cl) transporters, resulting in an overload-salt state that increases blood pressure (BP). Contrary to previous studies that have pointed out the direct linkage between fructose intake and hypertension, Bezerra et al. [5] showed that fructose only is not responsible for BP increase, but merely caused salt-sensitive hypertension, which would be chronic, when coupled with high-salt diet. Carbal et al. [7] and Gonzalez-Vicente et al. [8] demonstrated that consuming fructose coupled with a high-salt diet enhanced the sensitivity of the proximal tubule transport to angiotensin II, leading to hypertension. Singh et al. [9] showed that Cl accelerated fructose-induced hypertension. In addition, Gomes et al. [10] found that the chronic exposure of rats to a high-salt diet for 12 weeks led to a significant increase in their BP.

Hypertension, also named as “silent killer”, affects around one billion individuals worldwide, and causes more than nine million deaths yearly [11]. Its prevalence is predicted to be increased, affecting a total of 1.56 billion people in 2025 [11]. Hypertension is a significant risk factor that disposes sufferers to coronary heart problems, atherosclerosis, thrombosis diseases, and renal failure [12]. Pharmacological commercially available anti-hypertensive drugs, which belong to the angiotensin-I converting enzyme (ACE)-inhibitory class, such as Captopril, Enalapril, Lisinopril, etc., are often associated with various adverse side effects [13]. Therefore, novel, safe and efficient therapeutic agents are required for better management of hypertension.

Among natural antihypertensive compounds is the class of food proteins that contains active peptide fragments, encrypted within their structure and released upon their consumption and digestion with gastrointestinal enzymes to exert their beneficial effects in the organism [14]. These active peptide fragments have been previously described for their beneficial effect on hypertension management. In fact, marine peptides from *Salmo salar* [15] and *Capro saper* [16] were found to be efficient in reducing the BP of rats during a short period of time (within 24 h). On the other hand, the long-term antihypertensive effect of *Okamejei kenojei* peptides on reducing the BP, following their administration to rats for 20 days, was also proved [17]. In addition, peptides with molecular weight (MW) below 2 kDa, isolated from squid skin gelatin hydrolysate and orally administrated to hypertensive rats for 30 days, were able to remarkably reduce arterial BP [18]. Zhuang et al. [19] assessed the antihypertensive effect of long-term (30 days) oral administration of jellyfish collagen peptides on renovascular hypertensive rats. All of these peptides induced antihypertensive effect mainly through the inhibition of the ACE, the enzyme playing a crucial role through the renin-angiotensin system, the key system of BP regulation in human body. 

In a previous study, Abdelhedi et al. [20] produced peptides, gifted with interesting ACE-inhibitory activity, from smooth hound (*Mustelus mustelus*) viscera hydrolysates. In the present study, we aim to evaluate the therapeutic effect of these peptides against hypertension complications. Therefore, the anticoagulant and anti-lipase effects in vitro were first evaluated. Then their ability to reduceBP, restorelipids disorder, and prevent renal and hepatic toxicity, in vivo, were assessed using high-salt and -fructose diet-fed rats as animal models.

## 2. Results and Discussion

### 2.1. Protein Hydrolysate Preparation, Amino Acids Composition and Angiotensin-I Converting Enzyme (ACE)-Inhibitory Activity

Smooth hound viscera protein hydrolysate (VPH) prepared following enzymatic hydrolysis, using Purafect^®^ with a final degree of hydrolysis of about 14.5% [21], was used in the present study. VPH was fractionated through successive membrane filtration (ultra-filtration) steps to obtain peptides with MW below 1 kDa (VPH-I). Since the biological activities of peptides strongly depended on several factors, including their amino acids (AA) composition and sequences [22], the AA compositions of VPH and VPH-I were determined and compared to the intact (un-hydrolyzed) viscera proteins (UVP), as shown in Table 1. Data demonstrated that all the samples were mainly composed of Gly, Glx, Lys and Asx, followed by Arg, Pro and Ala. Although VPH contained a great amount of essential (His, Thr, Val, Met, Ile, Leu, Phe, Lys) and hydrophobic (Ala, Pro, Tyr, Val, Met, Ile, Leu, Phe) amino acids compared to the intact proteins, its derived fraction (VPH-I) showed the highest levels, reaching, respectively, 34.37% and 40.35%. This finding indicates that the fractionation of VPH through membrane separation led to concentrate high-quality amino acids in the retained fraction (VPH-I) and therefore improving its bioactivity and nutritional value. In fact, it has been demonstrated that hydrophobic residues and charged side groups have the greatest influence on the ACE-inhibitory potential. Sun et al. [23] reported that sequences with hydrophobic and positively charged C-terminal residues (Lys and Arg) showed an increased ACE-inhibitory activity. In addition, it has been demonstrated that the transport mechanism of ACE-inhibitory peptides is affected by their AA composition. For instance, Shimizu et al. [24] demonstrated that hydrophobic peptides consisting of 4 to 9 amino acids can passively diffuse through cell membranes by transcytosis or by para-cellular diffusion. These hypotheses expected the bioavailability and the bioactivity of VPH-I under physiological conditions.

In agreement with the amino acids composition results, VPH and VPH-I which contained the highest amount of HAA, revealed the highest ACE-inhibitory activity by 70% and 83% at 0.25 mg/mL, respectively (Supplementary Material), with respective IC_50_ of 75 ± 0.1 µg/mL and 53.31 ± 1.93 µg/mL, while it was of 731.22 µg/mL for UVP [20,21]. In addition, the high HAA content was in line with the future bioavailability of peptides, once consumed. In fact, it has been demonstrated in a previous study that the bioactivity of smooth hound peptides, particularly those rich in hydrophobic residues, remained constant and even increased after their simulated gastrointestinal digestion in vitro [21].

### 2.2. Anti-Pancreatic Lipase Activity In Vitro

**Figure 1:**
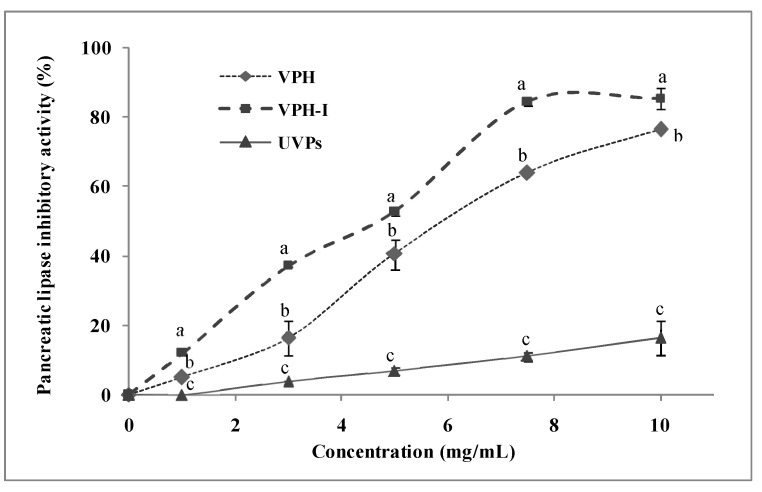
Pancreatic lipase inhibitory activity in vitro of UVP, VPH and VPH-I at different concentrations. Different letters in the same concentration within different samples indicate significant differences at *p* ≤ 0.05.

The inhibition of pancreatic lipase and ACE activities, are involved in the slowing-down of chronic metabolic disorders. Particularly, pancreatic lipase is involved in the hydrolysis of dietary fats and phospholipids and in the metabolic processing of LDL and HDL-cholesterol, besides its role in the absorption of gastrointestinal triglycerides [25]. Thus, the inhibition of the pancreatic lipase activity may be an effective pathway for the treatment of dyslipidemia. The inhibition of the pancreatic lipase activity by the different samples is depicted in Figure 1. At 5 mg/mL, the enzyme activity was significantly inhibited by about 41% and 53%, by the addition of VPH and VPH-I, respectively, using 4-MU oleate as substrate. A concentration of 7.5 mg/mL of these samples was sufficient to inhibit the lipase activity by 64% (VPH) and 85% (VPH-I). UVP showed, however, the lowest activity to reach its maximum of 16% at 10 mg/mL. Similar findings were reported by Shin et al. [26] for *Alpinia officinarum* extract, showing interesting inhibition of lipase activity, with a half maximal (50%) concentration (IC_50_) of about 3 mg/mL, using triolein as substrate, and an IC_50_ = 5.6 mg/mL, using tributyrin as substrate. However, these values were still lower than that of Orlistat (standard inhibitor of pancreatic lipase) showing an inhibition value of about 85% at 250 µg/mL.

### 2.3. Anti-Coagulant Activity

Huang et al. [27] demonstrated the strong link between hypertension and venous thrombosis, where this health risk increased 4 times in individuals with dyslipidemia [28]. Thus, in order to predict the protecting effects of VPH and VPH-I from thrombosis, samples were assayed for their ability to extend blood clotting times. To this end, activated partial thromboplastin time (aPTT), prothrombin ratio (PR) and thrombin time (TT) assays, the main clinical tests used to explore the intrinsic, extrinsic and common coagulation pathways, respectively, were investigated. 

#### 2.3.1. Activated Partial Thromboplastin Time (aPTT)

**Figure 2:**
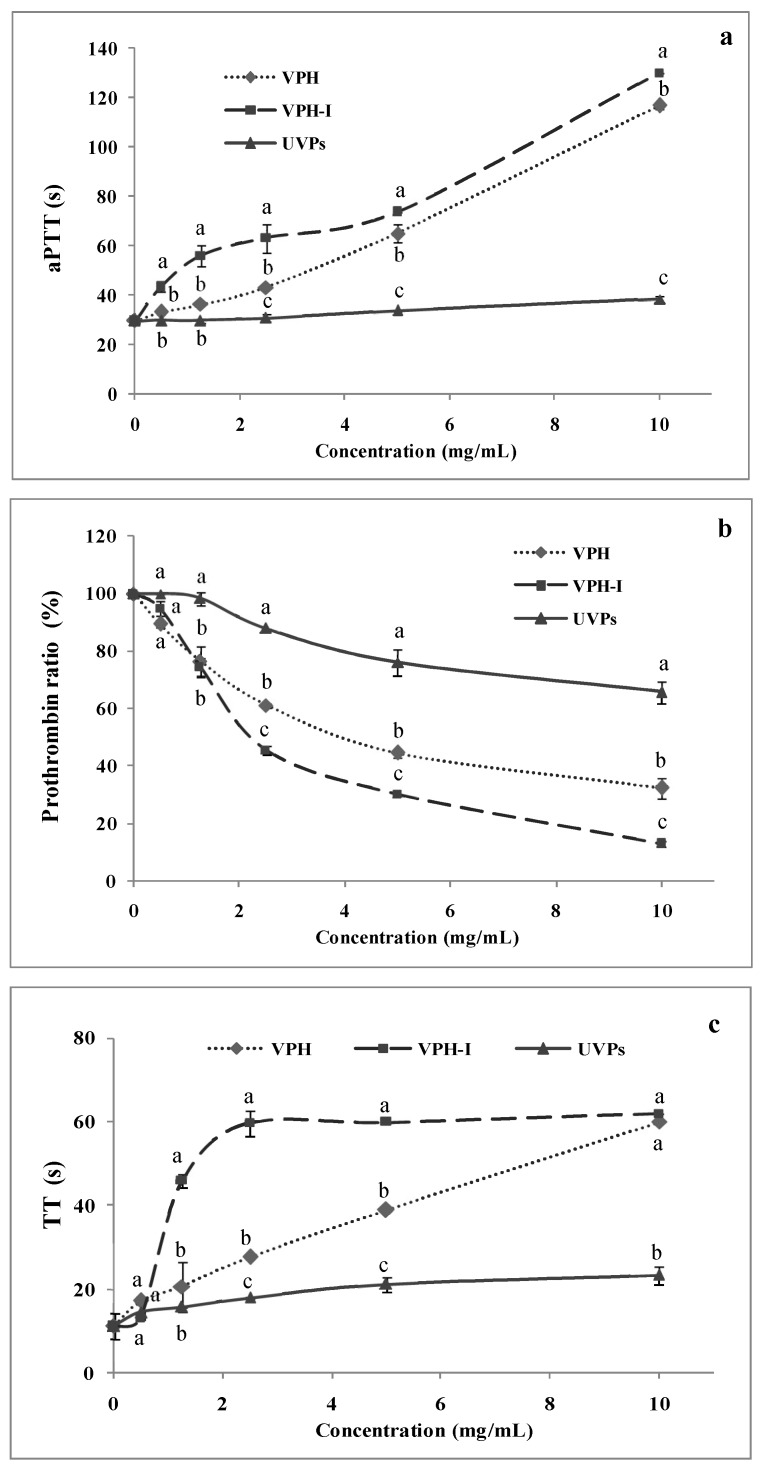
Effect of UVP, VPH and VPH-I on blood clotting time through (**a**) the activated partial thromboplastin time (aPTT), (**b**) the prothrombin ratio (PR) and (**c**) the thrombin time (TT). Different letters in the same concentration within different samples indicate significant differences at p ≤ 0.05.

The effects of UVP, its hydrolysate and peptides’ fraction on the aPTT at different concentrations, are illustrated in Figure 2a. The results clearly showed that, except UVP, the anticoagulant activity was concentration-dependent for all the samples. UVP was inefficient on prolonging the clotting time, whatever the concentration tested, and values still similar to the control normal blood (aPTT = 33 s). However, VPH-I was found to be the most effective on prolonging aPTT (*p* ≤ 0.05). In fact, a marked clotting time increase was obtained, exceeding 70 s and 120 s after the addition of 5 mg/mL and 10 mg/mL of VPH-I, respectively. VPH showed a slight lower activity compared to VPH-I, mainly due to the synergistic effect between peptides present in the same hydrolysate. Similar findings have been reported by Nasri et al. [29] for goby protein hydrolysate, obtained by the action of *Bacillus licheniformis* proteases, capable of delaying the clotting time 3-fold, compared to the undigested proteins, at a concentration of 30 mg/mL. In addition, Kong et al. [30,31] have isolated anticoagulant peptide sequences (TNGYT and SQL) from *Scolopendra subspinipes* able to extend the aPTT to 57 s and 71s at 8 mg/mL and 1 mg/mL, respectively.

#### 2.3.2. Pro-Thrombin Ratio (PR)

The pro-thrombin time, expressed also as pro-thrombin ratio (PR), of blood after UVP, VPH and VPH-I addition was evaluated (Figure 2b). As shown, similar to the aPTT results, VPH-I exhibited the highest anticoagulant effect providing a 50% pro-thrombin formation inhibition (IC_50_) at a concentration of 2.3 mg/mL, while the IC_50_ of VPH was about 4.8 mg/mL. As expected, UVP did not show a great potential, reaching its maximal of 34% at 10 mg/mL. In this context, Thakur et al. [32] isolated a new peptide sequence (MW = 4423.6 Da) from *Daboia russelii russelii* venom exhibiting a potency equivalent to that of heparin and able to prolong the prothrombin time in a dose-dependent manner.

#### 2.3.3. Thrombin Time (TT)

The human blood coagulation cascade consists of intrinsic and extrinsic pathways, which converge both to the common route, in which occurs the formation of factor (F) Xa, activated following a proteolytic digestion of FX, either by factor IXa (intrinsic pathway) or factor VIIa (extrinsic pathway). The measurement of the thrombin time (TT) allows an overall assessment of insoluble fibrin (FIa) formation, cleaved by the action of thrombin (FIIa) and representing the end-product of the clotting cascade [33]. Previous anticoagulant marine peptides have been reported as specific inhibitors of one factor among those involved in the coagulation mechanism [33]. The results of the TT of all samples as a function of their concentrations are presented in Figure 2c. The protein hydrolysate and its peptide fraction showed higher anticoagulant activity, compared to the intact proteins (about 13 s). In fact, VPH and VPH-I exhibited exponential activity shape, with a dose-dependent manner and showed 2-fold and 4-fold lengthening levels of blood clotting time, respectively, at 1.25 mg/mL. At a dose of 2.5 mg/mL, VPH-I induced the maximal extension time detected by the STart^®^ analyzer (60 s), indicating its effectiveness on inhibiting the common coagulation route. In the same regard, Jung and Kim [34] isolated an anticoagulant peptide (MEAP), from *Mytilus edulis*, able to prolong the aPTT and TT clotting times, but not PT. The aPTT was, in this study, delayed from 35 to 321 s and the TT from 11 to 81 s.

Regardless of the test used, the present investigation indicated that VPH-I exhibited the highest prolonging effect of blood clotting time, suggesting therefore its ability to interact with the clotting factors involved in the intrinsic, extrinsic and/or common coagulation cascade. In addition, the ineffectiveness of UVP demonstrated that the anticoagulant peptides were encrypted within the protein parent and then released after Purafect-proteolysis and ultra-filtration (UF) concentration.

### 2.4. In Vivo Analyses 

#### 2.4.1. Body Weight Gain, Food and Water Intake

**Figure 3:**
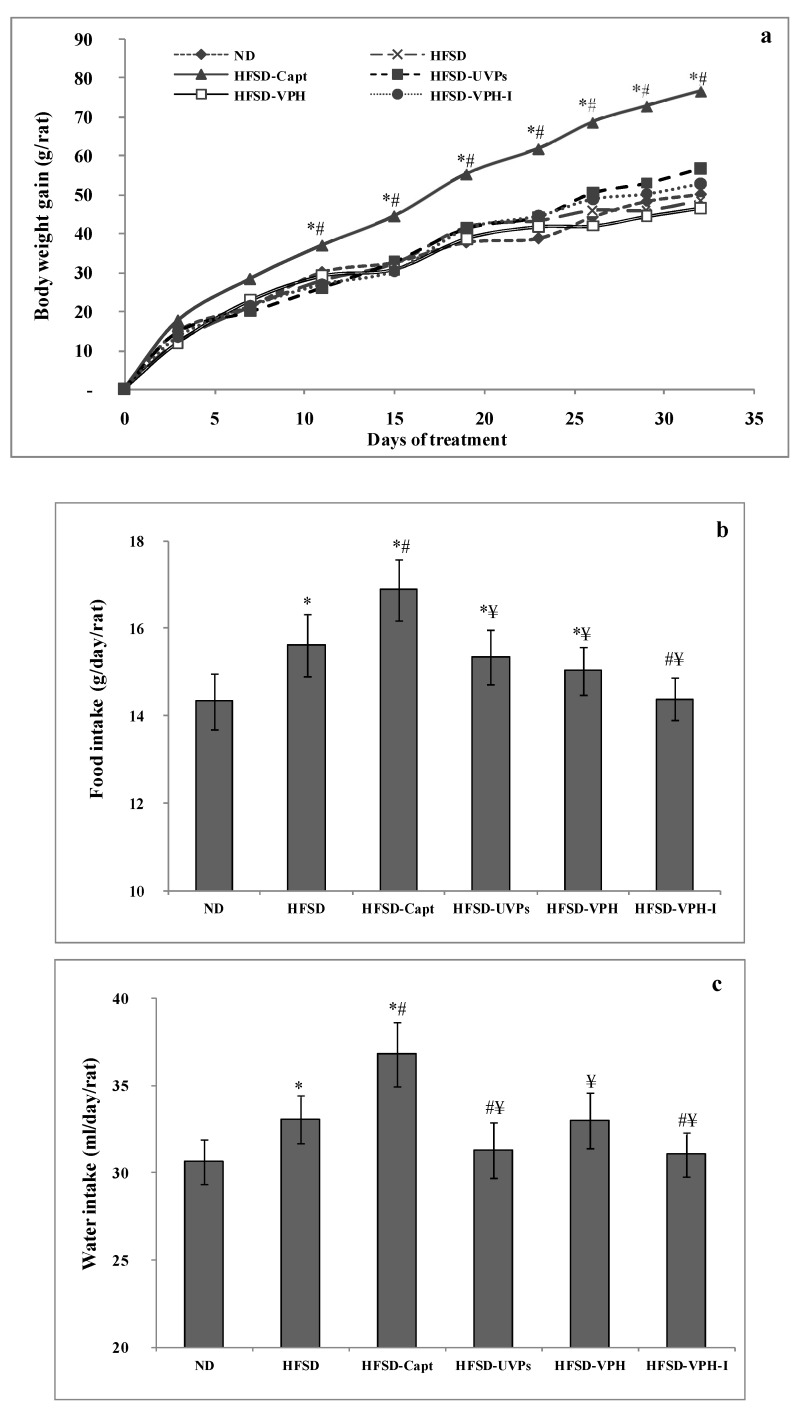
(**a**) Body weight gain, (**b**) food and (**c**) water intake of the different groups of rats through and after the experimental period. Symbols (*, # and ¥) indicate significant differences compared to the normal diet (ND), HFSD and HFSD-Capto groups, respectively (*p* ≤ 0.05).

During the period of the experiment, it is worth noting that the behavior of the animals was normal, indicating that the gavage of the different samples did not affect their psychological state. The body weight (BW) gain (g/rat) of all the animals was measured in a regular interval of time (each 4 days) and the results are reported in Figure 3a. As shown, except for high -salt and -fructose diet (HSFD)-fed rats and treated with Captopril (HFSD-Capt), all the animals exhibited similar BW increase, whatever the diet and the treatment received, showing values similar to the BW gain of the normal diet (ND) rats. Meanwhile, HFSD-Capt animals showed a significant overweight (*p* ≤ 0.05), compared to all the other groups, and their mean BW gain reached 77 g/rat after one month of treatment, which suggested that the treatment with the anti-hypertensive drug stimulated the appetite behavior of rats and, therefore, provided higher calorie intake compared to the other treatments. This hypothesis was confirmed by the food consumption level of all the groups during the experience. Indeed, data presented in Figure 3b indicated that HFSD-Capt animals consumed the highest level of diet, evaluated at 16.89 g/day/rat, compared to 14.33 g/day/rat and 15.62 g/day/rat in the ND and HFSD groups, respectively. These data indicated that Captopril may be also an appetite stimulator agent. However, among the viscera proteins-based treatments, only VPH-I was able to reduce the food intake to values similar to those of the normal rats, may be due to the satiating property of these peptides. 

To assess the effect of salt consumption on the thirstiness of animals, water intake was evaluated daily and the mean values related to each group of rats were calculated and presented in Figure 3c. Data indicated that, even after the daily oral gavage of 18% NaCl solution, HFSD-UVP, HFSD-VPH and HFSD-VPH-I animals consumed water with volumes of 31.32, 33.03 and 31.07 mL/day/rat, respectively, similar to the ND rats (30 mL/day/rat) and lower than the HFSD animals. However, similarly to the food intake, HFSD-Capt group showed the highest drinking water level estimated as a volume of 36.8 mL/day/rat. These results indicated that, contrary to the viscera peptides (200 mg/kg BW/day), the antihypertensive drug (20 mg/kg BW/day) was not efficient at reducing the thirstiness of the animals receiving a high quantity of salt. Similar results were reported by Bopda et al. [35], who induced hypertension by oral administration of salt, and showed that *Kalanchoe pinnata* leaf extract (100 mg/kg BW/day) dropped the water consumption of rats to 12 mL/day/rat, compared to 31 mL/day/rat in the untreated animals.

#### 2.4.2. Short- and Long-Term Antihypertensive Effect of Viscera Protein Hydrolysate (VPH) and VPH-I

**Figure 4:**
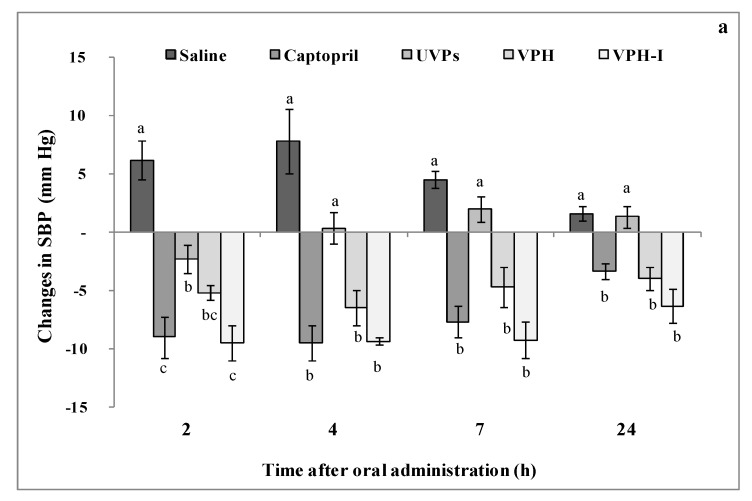

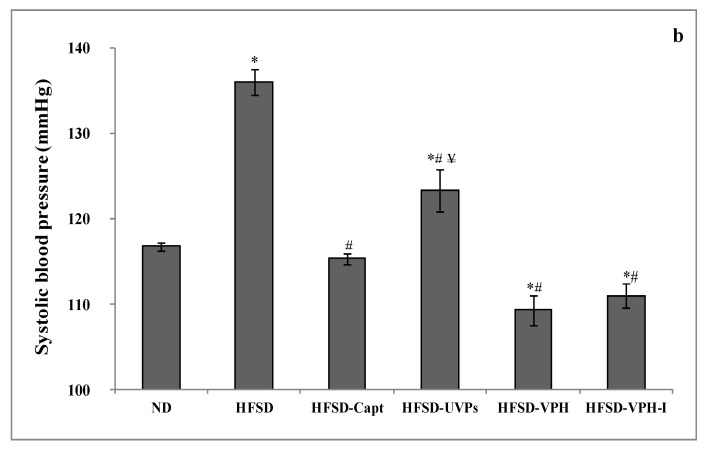
Changes in systolic blood pressure (SBP) of the different treatments following their (**a**) short- and (**b**) long-term administration. Different letters in Figure 4a indicate significant differences between groups at *p* ≤ 0.05 (n = 4). Symbols (*, # and ¥) in Figure 4b indicate significant differences compared to the ND, HFSD and HFSD-Capt groups, respectively, at *p* ≤ 0.05(n = 4).

The antihypertensive effect of all the treatments was evaluated by measuring the changes in systolic blood pressure (SBP) of rats, in short- and long-term experiments. Figure 4a represents the short-term changes in SBP of adrenaline-induced hypertensive rats measured during 24 h. Rats received by oral gavage 1 mL of saline solution, 20 mg Captopril/kg BW and 200 mg of UVP, VPH, or VPH-I per kg of BW. Differences in SBP (mmHg) were recorded after 2, 4, 7 and 24 h following each treatment administration, and compared to the initial SBP level (t = 0 min, before gavage). Results showed that the saline increased significantly the SBP of adrenaline-treated rats up to 24 h following its administration (*p* ≤ 0.05), where the maximum SBP increase was obtained after 4 h (+8 mmHg). The administration of UVP did not exhibit a marked antihypertensive effect, where the SBP values still similar to those of the saline control group, although the slight decrease recorded during the first 4 h. Nevertheless, the highest SBP-lowering effect obtained with VPH was about 4 mmHg at 24 h-post administration, whereas it was of 6 mmHg for VPH-I after the same interval of time. These effects were similar to that obtained with Captopril treatment, where the highest SBP reduction reached about 9 mmHg at t = 4 h. Similarly, Girgih et al. [14] have shown that salmon protein hydrolysate (200 mg/kg BW) and its most active ACE-inhibitory peptide fraction (30 mg/kg BW) reduced the SBP after 24 h of their oral administration to hypertensive rats.

On the other hand, the long-term antihypertensive effect was assessed by measuring the changes in SBP, 30 days after the daily oral administration of UVP, VPH or VPH-I (200 mg/kg BW) and Captopril (20 mg/kg BW) to rats fed a high salt and fructose diet (Figure 4b). The HSFD control group was given the same volume of saline. As shown, untreated HFSD group exhibited the highest SBP level among all the groups (136 mmHg). This increase (+17%) indicated that feeding rats with fructose/salt diet provided the development of hypertension state, as previously published by Klein and Kiat [6]. The authors showed that high sugar/high salt combination increased steadily the SBP to 132 mmHg. In fact, increased salt intake increased the rate of renal salt excretion, resulting in volume expansion and, as a consequence, hypertension [36].

Interestingly, over a 4-week treatment period, the oral administration of Captopril, VPH, or VPH-I clearly decreased SBP by 15%, 19% and 18%, compared to the HSFD-fed rats, reaching 115, 109 and 111 mmHg (*p* ≤ 0.05), respectively. These values were similar to that measured in the ND group (SBP = 117 mmHg). Even its marked BP decrease, UVP-treated rats showed, however, values significantly higher than those of the ND group. The maximal SBP decrement was recorded in HFSD-VPH and HFSD-VPH-I, indicating that smooth hound peptides exhibited a substantial effect on reducing hypertension in HFSD-model system during the long-term treatment. These findings are in accordance with the in vitro anti-ACE results, where the small peptides were the most active against ACE activity. The slight difference noted between VPH and VPH-I treatments may be due to the synergistic effect existing between peptides in the hydrolysate mixture. 

Similar results have been reported by Ngo et al. [14] for skate (*Okamejei kenojei*) skin gelatin peptides (below 1 kDa). In their study, the authors mentioned that SBP of hypertensive rats (~180 mmHg) decreased after the treatment with gelatin peptides (1000 mg/kg BW) until 132 and 127 mmHg at days 10 and 20, respectively. In addition, Zhuang et al. [19] demonstrated that the oral administration of jellyfish collagen peptides (100 mg/kg BW) for one month reduced the SBP from 190 to 135 mmHg in renovascular hypertensive rats, by reducing the angiotensin II concentration in rats’ kidney.

#### 2.4.3. Lipase Activity in Plasma

**Figure 5:**
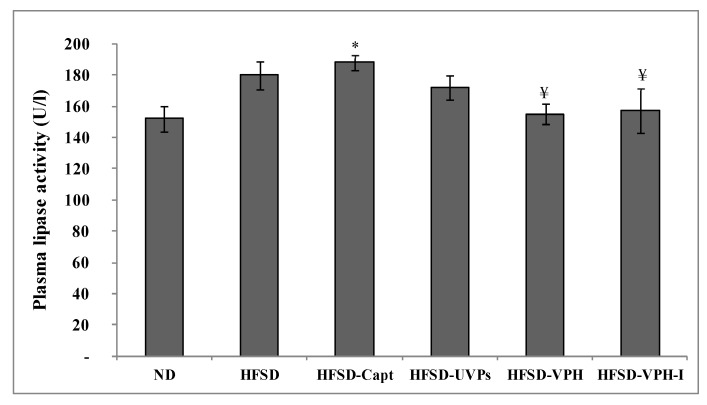
Plasma lipase activity of the different groups of rats at the end of the experimental period. Symbols (* and ¥) indicate significant differences compared to the ND, HFSD and HFSD-Capt groups, respectively (*p* ≤ 0.05).

After about four weeks of HFSD feeding, rats were sacrificed and the lipase activity in plasma was evaluated (Figure 5). As shown, the lipase activity in HFSD rats increased by 18.42%, compared to the control group (ND). However, the administration of the antihypertensive drug significantly increased this activity more than value recorded in the HFSD group (*p* ≤ 0.05), reaching 188 U/mL. This finding is in line with the BW gain and food intake results; in fact, HFSD-Capt rats showing the highest corporal mass and calories uptake, displayed the highest lipase activity. Increased lipase activity led to increase dietary fats and phospholipids hydrolysis, as well as triglycerides absorption, inducing, therefore, dyslipidemia [25]. However, the treatment with VPH and VPH-I decreased lipase activity from 180 U/mL in HFSD-fed rats to about 155 and 157 U/mL, respectively. By contrast, UVPs were not efficient on reducing the enzyme activity (172 U/mL). These results are in accordance with the in vitro findings of the lipase activity inhibition levels and demonstrate once again the importance of the viscera protein hydrolysates on preventing dyslipidemia. Similar results were reported by Nasri et al. [37] who showed that goby protein hydrolysates were able to reduce the lipid accumulation by the inhibition of the lipase activity in vivo, using hypercaloric diet-fed rats. 

#### 2.4.4. Lipid Profile and Athrogenic Index of Plasma

The levels of serum lipids in the different animal groups at the end of the experience are illustrated in Table 2. As shown, HFSD increased significantly (*p* ≤ 0.05) the levels of plasma total triglycerides (TG) and total cholesterol (TC) by approximately 37% and 62%, respectively, and decreased the HDL-c level by 19%, compared to the ND group. On another hand, Captopril administration increased lipids level in serum, particularly the TC content, reaching 3.45 mmol/L, value higher than that recorded in the HFSD (2.03 mmol/L). This effect may be due to the high energy intake of Captopril-treated rats, as shown in their food consumption level and BW gain, recorded during the experience. 

However, the daily administration of viscera-based proteins improved the lipids’ profiles of the treated animals, revealed by a decrease of TC and TG levels, as well as an increase in HDL-c level, in comparison with HFSD. In fact, VPH and VPH-I decreased the levels of TC to 1.68 and 1.83 mmol/L, respectively, compared to 2.03 mmol/L in HFSD group. The TG concentration was, similarly, reduced after VPH (0.97 mmol/L) and VPH-I (1.03 mmol/L) administration, while it was of 1.26 mmol/L in the untreated HFSD-fed animals. Moreover, HDL-c level showed corrected values achieving 0.78 ± 0.02 and 0.90 ± 0.02 mmol/L, in HFSD-VPH and HFSD-VPH-I groups, respectively, while values were markedly reduced in HFSD rats (0.56 ± 0.05 mmol/L). Although the oral gavage of UVP showed a hypolipidemic effect, by reducing the TG and TC levels to 1.02 and 1.90 mmol/L, respectively, it was less efficient than its hydrolysate (VPH) and peptide fraction (VPH-I), demonstrating the importance of the Purafect-protein hydrolysis. Also, it was noted that the hypolipidemic effect of VPH was greater than VPH-I, and this may be due to the presence of different peptide lengths (not only with MW ≤ 1 kDa) that work in synergy in the whole hydrolysate, resulting, therefore, in the observed increased activity.

The excess of TG and TC, associated with the low level of HDL-c, promotes the accumulation of lipid in the artery, and thereby increases the thrombosis risk. Thus, the atherogenic index of plasma (AIP) was calculated, as shown in Table 2. As expected, the AIP of the HFSD animals exhibited the highest value, reaching 0.34 vs. 0.12 in normal rats (Table 2). Meanwhile, the reduction of plasma lipids in treated rats and the improvement of HDL-c concentrations contribute to prevent the animals from cardiovascular disease risk by restoring the AIP. Indeed, the AIP values decreased after VPH and VPH-I treatments to achieve values similar to those of the ND rats, and significantly lower than the HFSD group (*p* ≤ 0.05). The cardio-protective effect of viscera peptides may be also strengthened by their anticoagulant activity, previously demonstrated in vitro. These results are consistent with those published by Nasri et al. [37], dealing with the administration of goby fish peptides, gifted with anticoagulant and antilipidemic activities, and capable to prevent hypercaloric rats from atherogenic and coronary diseases.

#### 2.4.5. Renal and Hepatic Parameters in Plasma

An increased salt intake increases the risk of salt-sensitive hypertension, which in turn induces abnormalities of kidney function by increasing NaCl reabsorption, decreasing glomerular capillary filtration coefficient, or causing nephron injury [38]. To assess the effect of HFSD-induced hypertension on the kidney function of rats, urea, creatinin and uric acid levels were evaluated, as illustrated in Table 2. Urea and uric acid are among the breakdown products resulting from the nucleic acids’ metabolism. The plasma levels of creatinin and uric acid represent excellent indicators about renal function (glomerular filtration rate). As shown, there was no significant difference in the levels of uric acid and creatinin between ND and HSFD-fed rats, suggesting that the animals did not suffer from renal failure after the consumption of HFSD. 

Although a high level of uric acid is strongly associated with many diseases, such as kidney dysfunction, development of hypertension, insulin resistance, dyslipidemia, etc., uric acid is a powerful scavenger of free radicals providing about 60% of free-radicals scavenging in plasma [39,40]. On the other hand, Nishida [41] demonstrated that accelerated uric acid synthesis was associated with increased creatinin synthesis, which is in line with the observed increased levels in uric acid and creatinin in VPH-I treated rats. Thus, it is possible to suggest that the observed slight increase in plasma levels of uric acid and creatinin in VPH-I treated rats represent an adaptive physiological response of rats to be protected against excessive production of free radicals and oxidative stress-caused by HSFD. However, Captopril and UVP treatments induced a significant reduction of these values (*p* ≤ 0.05), compared to the ND.

For the hepatic biomarkers, data showed no significant differences between all the groups for AST, ALT and ALP activities, as well as the total bilirubin level, except some small variations noted may be due to the experimental conditions (Table 2). The present data indicated the healthy liver function of all the rats, whatever the treatment received and although the HFSD feeding. Similar findings have been reported by Jemil et al. [42] showing that the daily gavage of sardinelle protein hydrolysates and the hypercaloric diet (fat and fructose) did not induce liver inflammation, as revealed by the activities of AST and ALT in the different animal groups.

#### 2.4.6. Plasma Glucose Level

**Figure 6:**
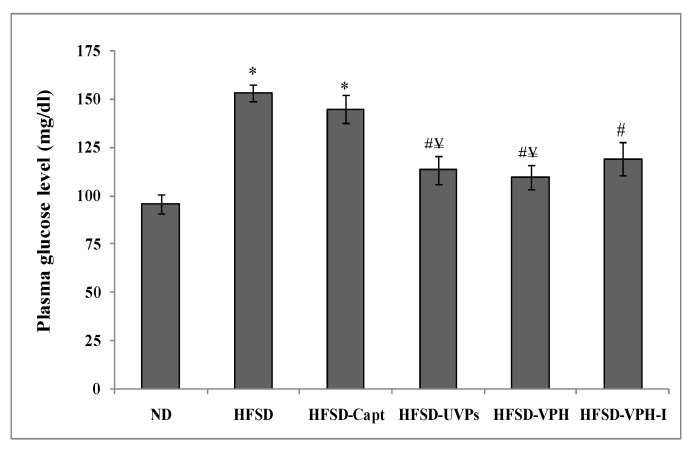
Plasma glucose level of the different groups of rats at the end of the experimental period. Symbols (*, # and ¥) indicate significant differences compared to the ND, HFSD and HFSD-Capt groups, respectively (*p* ≤ 0.05).

The plasma glucose level was evaluated in the different rats’ groups after four weeks of HFSD administration, and the values are shown in Figure 6. Data demonstrated that HFSD increased significantly the level of plasma glucose, when compared to the control diet (153.31 ± 4.31 mg/dL vs. 95.56 ± 4.87 mg/dL) (*p* ≤ 0.05), which proves that the high fructose diet induced hyperglycemia in rats. In line with this finding, it has been shown that high fructose diet (fructose instead of corn starch for 8 weeks) induced fasting hyperglycemia, hyper-insulinemia and glucose intolerance [43]. However, the oral administration of VPH and VPH-I reduced the plasma glucose levels by 28.5% and 22.5%, respectively, compared to HFSD group, proving the anti-hyperglycemic potential of viscera peptides. Similarly, UVPs were found to reduce slightly the plasma glucose level, but values still higher than that of the control ND group. To note, Captopril treatment was not efficient on blood glucose level reduction and animals showed values similar to that of the HFSD group (144.69 ± 7.40 mg/dL). In fact, Erlich and Rosenthal [44] demonstrated that ACE-inhibitory drugs (Enalapril, Lisinopril and Ramipril at 10 and 20 mg/kg BW/day), induced, in a dose-dependent manner, insulin resistance caused by high-fructose intake in rats, despite their antihypertensive effect. Interestingly, viscera peptides displayed, however, in vivo anti-hyperglycemic and antihypertensive activities. 

## 3. Materials and Methods

### 3.1. Biological Material

The biological material used for peptides’ elaboration is the viscera mass (stomach and intestine), obtained following the processing of fresh filleted smooth hound (*Mustelus mustelus*) fish, available in the local fish market of Sfax City, Tunisia. The raw material was brought to the research laboratory in iced conditions. Viscera were vigorously rinsed with tap water, then, immediately weighed and stored at −20 °C until use for protein hydrolysate preparation.

### 3.2. Animals 

Normotensive male Wistar rats weighing between 150–200 g were purchased from the Central Pharmacy of Tunis (SIPHAT, Tunisia). Animal maintenance was performed in accordance with the Guidelines for Care and Use of Laboratory Animals of Tunis University and approved by theDirective 2010/63/EU andthe Animal Ethics Committee of National Institute of Health [45]. Animals were kept in an environmentally controlled breeding room (temperature 22 ± 2 °C, relative humidity 60 ± 5%, 12 h dark/12 h light cycle) in the laboratory of the Faculty of Sciences of Sfax City, Tunisia. All rats were allowed free access to tap water and alimentation during the experimental period.

### 3.3. Protein Hydrolysate and Peptides Preparation 

Viscera from smooth hound fish were hydrolyzed as previously described [20], using Purafect^®^ (2000E), from *Bacillus licheniformis*. The degree of hydrolysis (DH) of the viscera protein hydrolysate (VPH), calculated using the method of Adler-Nissen [46], was found to be 14.5%. Undigested viscera proteins (UVP) were treated under the same conditions, without enzymes’ addition and serve as control [20]. All samples were freeze-dried (Bioblock Scientific Christ ALPHA 1-2, IllKrich-Cedex, France) and stored at −20 °C until use. Low MW peptides from VPH were recovered following successive ultra-filtration (UF) steps, as reported by Abdelhedi et al. [20], where the last peptide fraction (MW ≤ 1 kDa) was freeze-dried and referred to VPH-I.

### 3.4. Amino Acids Composition

The amino acids (AAs) composition was determined according to Aristoy and Toldrá [47], as the following. After being dried under nitrogen-vacuum cycles, samples were hydrolyzed, in vacuum-sealed glass tubes, in the presence of 300 µL of HCl (6 M) containing 1% (*v*/*v*) phenol, at 120 °C for 24 h. Thereafter, samples were derivatized with phenyl isothiocyanate (PITC) and dissolved in 5 mM sodium phosphate buffer (pH 7.4) containing 5% (*v*/*v*) of acetonitrile. The PITC derivates were quantified by a reversed phase chromatography (RP-HPLC) system (Agilent Technologies, Palo Alto, CA, USA), equipped with a PicoTag^®^ column (300 mm× 3.9 mm, Waters) and set at 52 °C. AAs’ detection was carried out at 254 nm. Norleucine was mixed with each sample, and treated under the same conditions, serving as internal standard. The AAs content was quantified compared to the amino acid standards and the results were expressed in g of amino acid per 100 g amino acids. 

### 3.5. Angiotensin-I Converting Enzyme (ACE)-Inhibitory Activity

The ACE-inhibitory (ACE-I) activity of samples was measured according to Sentandreu and Toldrá [48]. The sample solution (50 μL), prepared at different concentrations, was mixed with 50 μL of Tris-base buffer (150 mM, pH 8.3) containing 3 mU/mL of ACE solution. Then, 200 μL of buffer, containing 1.125 M NaCl and 10 mM o-aminobenzoyl-glycyl-p-nitro-l-phenylalanyl-l-proline (Abz-Gly-Phe-(NO_2_)-Pro), were added. The reaction mixture was then incubated for 60 min at 37 °C. The fluorescence was measured, each 15 min, during 1 h, using excitation and emission wavelengths of 355 and 405 nm, respectively. Captopril (Capt) was used as a positive control. The results were expressed as percentage of ACE inhibition, as a function of concentrations.

### 3.6. Pancreatic Lipase Inhibitory Activity In Vitro

The pancreatic lipase inhibitory activity was measured using 4-methylumbelliferyl oleate (4-MU oleate) (Sigma, St. Louis, MO, USA), as a substrate, as previously described by Zhang et al. [48]. A volume of 5 μL of sample solution, or Orlistat (Sigma, St. Louis, MO, USA) used as positive control, was mixed with 50 μL of 0.1 mM 4-MU oleate, 20 μL of 0.1 M citrate-Na_2_-HPO_4_ buffer (pH 7.4) and 25 µL of porcine pancreatic lipase. The mixture was then incubated at 37 °C for 10 min, and the fluorescence intensity of 4-MU, released by the lipase activity, was measured at excitation and emission wavelengths of 320 nm and 450 nm, respectively, using a fluoroskan micro-plate reader (Fluoroskan Ascent FL, Thermo Electron Corporation, Vantaa, Finland). The inhibition of pancreatic lipase activity was determined in percentage at different samples’ concentrations.

### 3.7. Anticoagulant Activity

Human whole blood was collected from healthy volunteers via veni-puncture into siliconized Vacutainer^TM^ tubes (Becton Dickinson, Le Pont de Claix, France), containing buffered sodium citrate.Blood was then treated to prepare the platelet poor plasma (PPP), serving for the coagulation assays, as described by Ben Mansour et al. [49]. The anticoagulant activity was conducted based on three assays: the activated partial thromboplastin time (aPTT), the prothrombin ratio (PR) and the thrombin time (TT). Analyses were performed using a semi-automatic line (STart^®^ analyzer, Diagnostica Stago, France). All samples were dissolved in physiological saline solution, and tested in triplicate.

#### 3.7.1. Activated Partial Thromboplastin Time

For the aPTT assay, 5 µL of sample solution, dissolved at various concentrations in physiological saline solution, was mixed with 45 µL of PPP. After being incubated for 3 min at 37 °C, 50 µL of aPTT reagent (CK-PREST^®^, Diagnostica Stago S.A.S, France) were added to the mixture and then incubated again for 3 min at 37 °C. Thereafter, 100 µL of CaCl_2_ solution (0.025 M) were added to initiate the reaction, and the blood clotting time, measured in seconds, was immediately recorded. For the control reaction, the clotting time was measured by substituting the sample by physiological solution. In the aPTT test, all measurements up to 120 s were not taken to be significant.

#### 3.7.2. Pro-Thrombin Ratio

In the prothrombin ratio, 45 µL of PPP were mixed with 5 µL of each sample solution and pre-incubated for 3 min at 37 °C. Then 100 µL of Neoplastin^®^ reagent, pre-heated at 37 °C, were added and the clotting time was measured. The prothrombin time was obtained in seconds and then converted into prothrombin ratio (PR, %). In the control tube, the physiological solution was used instead of sample. In the absence of an anticoagulant agent, PR = 100%.

#### 3.7.3. Thrombin Time

The thrombin time (TT) assay was carried out by mixing 10 µL of each sample solution with 90 µL of PPP. After being incubated for 3 min at 37 °C, 100 µL of thrombin (80 NIH) were added and the clotting time started to be recorded. The TT value was expressed in seconds, compared to the control PPP, deprived of sample. Values of TT exceeding 60 s were not taken as significant. 

### 3.8. Animals Diet, Hypertension Induction and Treatment 

#### 3.8.1. Short-Term Effect

After the acclimatization period, 0.25 mg of adrenaline (Adrenaline Renaudin, Itxassou, France) per kg of body weight (BW) was daily injected into rats through intraperitoneal route using a 1 mL syringe for one week to induce hypertension [50]. The daily systolic blood pressure (SBP) was evaluated to confirm hypertension induction. After one week, rats were divided into five groups of four animals each. Rats receiving 1 mL of saline solution (sodium phosphate-buffered saline (PBS, pH 7.4)) were taken as untreated negative control group, while those treated with Captopril (20 mg/kg BW diluted in PBS) served as the positive control group. The other three groups were treated with the UVP, VPH and VPH-I (200 mg/kg BW), to assess their short-term effect (24 h) on reducing blood pressure. All the treatments were diluted in the buffered saline and administrated by oral gavage using a metal gastric tube. The SBP was then measured at 2, 4, 7 and 24 h and changes in BP values (ΔSBP, mm Hg) were expressed at each time, compared to the baseline value (SBP at time zero, before treatment administration).

#### 3.8.2. Long-Term Treatment

After the acclimatization period, rats were divided into six groups of six animals each. The first group received tap water and normal commercial diet, serving as the normal animals’ diet (ND) group. The second one was named as high-salt and -fructose diet (HSFD)-fed rats, by the administration of 1 mL 18% NaCl solution/kg BW/day and the addition of 10% of pure fructose in their daily food. The 3rd group received HSFD and Captopril^®^ at a dose of 20 mg/kg BW (HSFD-Capt). The other three groups, named HSFD-UVP, HSFD-VPH and HSFD-VPH-I, received likewise HSFD coupled to the treatment with UVP, VPH and VPH-I (200 mg/kg BW/day), respectively. A volume of 1 mL of all the treatments, dissolved in 18% NaCl solution, was daily administrated by oral gavage for one month, in a specific interval of time (10.00–11.00 a.m.), using a metal gastric tube. During the experiment, BW, food consumption and water intake were assessed regularly. Day before the sacrifice, SBP (mmHg) of all the animals was determined by a non-invasive method using an automatic animal blood pressure system (BP-AccurGard^TM^, Vmed Technology, Animal Blood Pressure Solutions) to assess the long-term antihypertensive effect of all the treatments. 

At the end of the experimental period, rats were sacrificed by decapitation. The blood was then collected in heparin-containing tubes, and the plasma supernatant, recovered after a centrifugation step at 5000×*g* for 15 min, was stored at −80 °C for biochemical analysis.

##### Determination of Lipid, Kidney and Hepatic Parameters in Plasma

Total cholesterol (TC), triglycerides (TG), and high-density lipoprotein cholesterol (HDL-c) concentrations (mmol/L) in plasma of all the groups were determined by enzymatic colorimetric methods using specific commercial kits to each test (Beckman Coulter, Maryfort, O’callaghans Mills, Co. Clare, Ireland) on an automatic biochemistry analyzer (UniCel^®^DxC 600, Beckman Coulter) in the Habib Bourguiba hospital of Sfax City, Tunisia. To assess the cardiovascular state, the atherogenic index of plasma (AIP) was determined, as the following:

AIP = log_10_(TG/HDL-c)

As the same, analysis indicating about the kidney (plasma concentrations of urea, creatinin and uric acid) and the hepatic (aspartate amino transferase AST, alanine amino transferase ALT and alkaline phosphatase ALP activities and total bilirubin TB concentration) functions were performed using specific commercial kits (Beckman Coulter, Maryfort, O’callaghans Mills, Co. Clare, Ireland) on an automatic biochemistry analyzer (UniCel^®^DxC 600, Beckman Coulter) in the hospital of Habib Bourguiba of Sfax City, Tunisia.

##### Plasma Lipase Activity

The plasma lipase activity was evaluated using specific commercial kit (SYNCHRON LX^®^ Systems Enzyme Validator Set) on an automatic biochemistry analyzer (UniCel^®^DxC 600, Beckman Coulter) in the Habib Bourguiba hospital of Sfax City, Tunisia. The plasma lipase activity in each group was expressed in U/L.

##### Plasma Glucose Level Evaluation

The plasma glucose level in each group was determined by an enzymatic colorimetric method using a commercially available kit (Glucose Oxidase-PAP, Biomaghreb, Tunisia).

### 3.9. Statistical Analysis

Results were expressed as mean ± standard error mean (SEM) and analyzed using the Statistical software SPSS ver. 17.0 (Professional edition, SPSS Inc., Hong Kong, China). A one-way analysis of variance (ANOVA) coupled with Duncanand LSD (least significant difference) tests were done to estimate the significance among the effects at the 5% probability level (*p* ≤ 0.05). The following symbols (*, # and ¥) indicate significant differences compared to the ND, HFSD and HFSD-Capto, respectively.

## 4. Conclusions

The present study indicated that the daily intake of high-salt and -fructose diet induced severe metabolic disorders, demonstrated by the hypertensive and hyperglycemic states. Short- and long-term administration of VPH and its fraction VPH-I were found to attenuate hypertension mainly by inhibiting the ACE activity, besides their role in correcting the lipid disorder, reducing plasma glucose level and protecting from thrombosis. Overall, the present findings encourage further exploration of smooth hound viscera peptides for functional foods and nutraceutical applications.

## Figures and Tables

**Table 1:** Amino acid (AA) compositions of the smooth hound viscera protein hydrolysate (VPH) and its ultra-filtrated fraction (VPH-I), compared to the undigested viscera proteins (UVP).

AA	UVP	VPH	VPH-I
**Asx #**	7.77 ± 0.14 ^b^	9.33 ± 0.24 ^a^	7.92 ± 0.02 ^b^
**Glx #**	12.92 ± 0.23 ^b^	13.79 ± 0.35 ^a^	13.48 ± 0.03 ^a^
**Hyp**	6.18 ± 0.11 ^a^	3.20 ± 0.08 ^b^	1.75 ± 0.0 ^c^
**Ser**	4.45 ± 0.08 ^c^	4.93 ± 0.13 ^b^	5.37 ± 0.01 ^a^
**Gly**	16.12 ± 0.29 ^a^	11.39 ± 0.29 ^b^	11.49 ± 0.03 ^b^
**Tau**	4.64 ± 0.08 ^b^	4.34 ± 0.21 ^b^	6.18 ± 0.01 ^a^
**His**	0.92 ± 0.02 ^c^	1.05 ± 0.03 ^b^	1.31 ± 0.0 ^a^
**Thr**	4.87 ± 0.09 ^b^	5.69 ± 0.15 ^a^	5.94 ± 0.01 ^a^
**Ala**	6.53 ± 0.12 ^b^	6.10 ± 0.16 ^b^	7.17 ± 0.02 ^a^
**Arg**	7.82 ± 0.14 ^a^	7.29 ± 0.19 ^b^	6.21 ± 0.01 ^c^
**Pro**	7.36 ± 0.13 ^a^	5.99 ± 0.15 ^b^	5.08 ± 0.01 ^c^
**Tyr**	0.51 ± 0.01 ^c^	1.03 ± 0.03 ^a^	0.98 ± 0.0 ^b^
**Val**	0.21 ± 0.00 ^c^	3.88 ± 0.1 ^b^	4.17 ± 0.01 ^a^
**Met**	4.03 ± 0.07 ^a^	1.47 ± 0.04 ^c^	2.23 ± 0.01 ^b^
**Ile**	2.13 ± 0.04 ^b^	3.03 ± 0.08 ^a^	2.91 ± 0.01 ^a^
**Leu**	3.14 ± 0.06 ^c^	4.42 ± 0.11 ^b^	4.69 ± 0.01 ^a^
**Phe**	2.30 ± 0.04 ^b^	3.17 ± 0.08 ^a^	3.55 ± 0.01 ^a^
**Lys**	8.11 ± 0.15 ^b^	9.89 ± 0.25 ^a^	9.55 ± 0.02 ^a^
**HAA**	26.21	29.09	40.35
**EAA**	25.71	32.60	34.37
**TAA**	100	100	100

Results (%: g per 100 g of amino acids) are expressed as mean ± standard deviation (SD); UVP represents the undigested viscera proteins; VPH represents the smooth-hound protein hydrolysate prepared using Purafect^®^; VPH-I represents the ultra-filtration (UF) fraction peptides with molecular weights below 1 kDa; a,b,c: Different letters in the same line indicate significant difference (*p* ≤ 0.05); #Asx and Glx indicate Asp+Asn and Glu+Gln, respectively; Trp and Cys were not determined; HAA: hydrophobic amino acids (Ala, Pro, Tyr, Val, Met, Ile, Leu, Phe); EAA: essential amino acids (His, Thr, Val, Met, Ile, Leu, Phe, Lys); TAA: total amino acids.

**Table 2:** Effect of the different treatments on plasma lipid (TG, TC, HDL-C, AIP), renal (urea, creatinin, uric acid) and hepatic (AST, ALT, ALP, TB) parameters of the experimental groups of rats

	ND	HFSD	HFSD-Capt	HFSD-UVP	HFSD-VPH	HFSD-VPH-I
**TG ^1^**	0.92 ± 0.05	1.26 ± 0.17 *	1.19 ± 0.04 *	1.02 ± 0.04 ^#¥^	0.97 ± 0.04 ^#¥^	1.03 ± 0.14 ^#¥^
**TC ^1^**	1.25 ± 0.19	2.03 ± 0.53	3.45 ± 0.29 *^#^	1.90 ± 0.31 ^¥^	1.68 ± 0.25 ^¥^	1.83 ± 0.27 ^¥^
**HDL-C ^1^**	0.69 ± 0.02	0.56 ± 0.05 *	0.74 ± 0.05 ^#^	0.71 ± 0.04 ^#^	0.78 ± 0.02 ^#^	0.90 ± 0.02 *^#¥^
**AIP**	0.12 ± 0.02	0.34 ± 0.03 *	0.21 ± 0.03 *^#^	0.14 ± 0.01 ^#^	0.11 ± 0.01 ^#¥^	0.12 ± 0.02 ^#¥^
**Urea ^1^**	5.85 ± 0.21	6.10 ± 0.31	6.33 ± 0.26	5.75 ± 0.27	5.45 ± 0.18 ^¥^	5.44 ± 0.19 ^¥^
**Creatinin ^2^**	23.25 ± 1.25	23.50 ± 1.19	23.67 ± 1.50	24.50 ± 1.12	25.50 ± 1.19	27.25 ± 0.75
**Uric acid ^2^**	61.50 ± 8.34	60.0 ± 7.31	44.83 ± 2.17 *	43.60 ± 2.56 *	57.60 ± 1.33	66.50 ± 9.74
**AST ^3^**	224.50 ± 16.73	228.0 ± 40.07	201.33 ± 15.19	214.80 ± 12.34	217.80 ± 17.39	227.50 ± 20.15
**ALT ^3^**	63.60 ± 4.37	59.60 ± 1.03	60.05 ± 3.78	61.75 ± 3.45	61.50 ± 4.97	69.25 ± 3.57
**ALP ^3^**	206.10 ± 20.63	231.40 ± 16.31	224.80 ± 27.87	224.17 ± 11.62	233.17 ± 25.22	222.50 ± 26.45
**TB ^1^**	6.73 ± 0.67	6.17 ± 0.38	6.0 ± 0.32	6.83 ± 0.70	6.42 ± 0.38	6.53 ± 0.73

^1^:mmol/L; ^2^:µmol/L; ^3^: U/L; Symbols in the same line (*, # and ¥) indicate significant differences compared to the ND, HFSD and HFSD-Capt groups, respectively (*p* ≤ 0.05); TG: total triglycerides; TC: total cholesterol; HDL-C: high density lipoprotein-cholesterol; AIP: athrogenic index of plasma (AIP = log10 (TG/HDL-C)); AST: aspartate amino transferase; ALT: alanine amino transferase; ALP: alkaline phosphatase; TB: total bilirubin.

## Supplementary Materials

The following is available online at https://www.mdpi.com/1660-3397/17/4/194/s1. Figure S1: ACE-inhibitory activity of UVP, VPH and VPH-I at the different concentrations.
