# Supplementary material for: Assessment of Cholesterol, Glycemia Control and Short- and Long-Term Antihypertensive Effects of Smooth Hound Viscera Peptides in High-Salt and Fructose Diet-Fed Wistar Rats
Source: Mar Drugs. 2019 Mar 27;17(4):194. doi: 10.3390/md17040194 (PMC6520678; doi:10.3390/md17040194)

Supplementary material:

ACE-inhibitory activity of UVP, VPH and VPH-I at the different concentrations

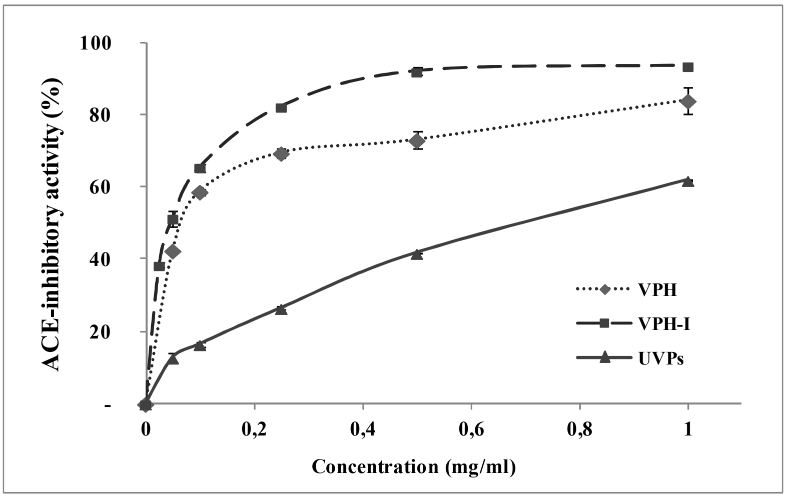

Supplement: Supplementary file 1 [file marinedrugs-17-00194-s001.pdf]
